# Size and charge correlations in spherical electric double layers: a case study with fully asymmetric mixed electrolytes within the solvent primitive model

**DOI:** 10.1039/d0ra06145j

**Published:** 2020-10-23

**Authors:** Chandra N. Patra

**Affiliations:** Theoretical Chemistry Section, Chemistry Group, Bhabha Atomic Research Centre Mumbai 400 085 India

## Abstract

Size and charge correlations in spherical electric double layers are investigated through Monte Carlo simulations and density functional theory, through a solvent primitive model representation. A fully asymmetric mixed electrolyte is used for the small ions, whereas the solvent, apart from being a continuum dielectric, is also treated as an individual component. A partially perturbative density functional theory is adopted here, and for comparison, a standard canonical ensemble Monte Carlo simulation is used. The hard-sphere free energy is treated within a weighted density approach and the residual ionic contribution is estimated through perturbation around the uniform density. The results from both methods corroborate each other quantitatively over a wide range of physical parameters. The importance of structural correlations is envisaged through the size and charge asymmetry of the supporting electrolytes that includes the solvent as a component.

## Abstract 2

Size and charge correlations in spherical electric double layers are investigated through Monte Carlo simulations and density functional theory, through a solvent primitive model representation.

## Graphical abstract

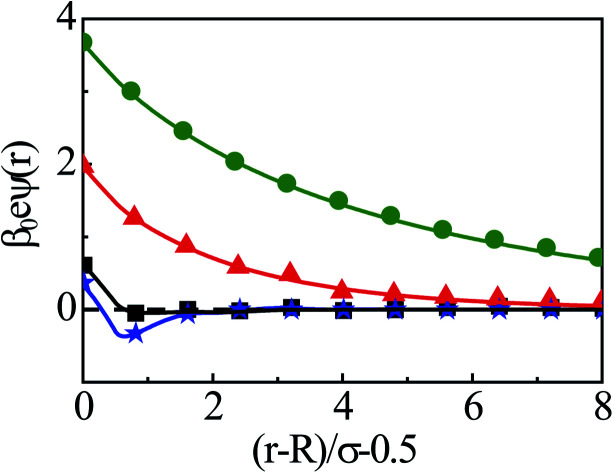

## I. Introduction

Modern day scientific and technological developments can be appreciated^1^ not only through the improved quality of life for mankind, but also through the miniaturization and productivity of devices, where the focus is mainly on energy, the environment and health and safety.^2^ The core research on such developments is based on the innovation of design, process development, delivery, and deployment.^3^ The process should be efficient, green or environmentally friendly, and intelligently controlled –requiring minimum human intervention, thereby giving rise to long term stability and flexibility in applications. Recent discoveries on a number of health related devices including nano-kits for first aid, nano-surgical equipment, and multiple user ventilators are a few of these developments that are helping humans continuously.^4^ Even personal protective equipment (PPE) and gear that requires specific protection against viruses and bacteria is also dependent on innovations in design and quality that define their robustness and usability.^5^ The organization of present day materials and their application versatility in the fabrication of devices requires thorough knowledge of bulk and interfacial states of matter to obtain the desired properties.^6^

Although understanding bulk states of matter is a past problem, current day fabrication routes of more complex materials still require accurate description of the bulk matter, along with the interfacial states for which phase diagrams are still in the fledging state. The multiscale modeling of materials and devices is a concrete step in this direction which allows a seamless transition from one scale to the other in terms of length and time.^7^ Also, suitable manipulation of the interface with a number of new synthesis strategies, as applied to nanomaterials, are presently in the advanced state of application.^8^ The intricate delicacies of the development^9^ of nanodevices, as applied to nanotechnological applications, requires new methods of fabrication, including the materials, the medium, and their associated interactions. A delicate modulation of such systems also requires a molecular level description so that the design and behavior can be formulated,^10^ even with a minute change of interaction.

The fact that interactions play a major role not only in the design but also in the fabrication of devices,^11^ can be directly visualized from a number of experiments that involve ions, radicals, dipoles, and even multipoles. Alongside the direct interactions between the molecular components, the medium^12^ or the associated solvent,^13^ also plays a major role^14^ in the modification of these interactions.^15^ For example, crowding effects,^16,17^ mostly observed in complex polymeric and biological macromolecular solutions, arise due to the presence of the solvent molecules. Similarly, solvents also act as a stabilizing medium for the ground or excited states for a number of molecules giving rise to different spectrochemical properties known as solvatochromic shifts.^18^ It also manifests the caging effects,^19^ for ions, radicals, and polar molecules, giving rise to a slowing down in solvation time correlation functions. In order to have a direct visualization of the qualitative and quantitative aspects of charge and size correlations arising due to ionic interactions and that of the solvent,^20^ in the current work, an attempt is made to understand both the effects in the same system, *viz.* in colloidal solutions where the supporting electrolyte and solvent is present as individual components.

Although, work including a molecular description of the solvent in an electric double layer (EDL)^21,22^ in planar,^23^ cylindrical,^24^ and spherical geometry,^25^ has been reported previously, these are mainly restricted within the restricted primitive model (RPM),^21^ mainly because of its computational simplicity. However, these studies could reveal important findings related to the volume exclusion introduced through the solvent as the competition between energetic and entropic effects gives rise to overcharging (OC)^26^ and charge reversal (CR)^26^ phenomena in such EDLs. Attempts to include the size asymmetry of the component ions as well as the solvent within the primitive model (PM) have been studied quite extensively in recent times.^25,27,28^ However, they are also mostly confined within the system of binary electrolyte that includes the solvent^29^ or a mixed electrolyte system without solvent.^30^ Although, effects of size asymmetry and charge asymmetry on EDLs are understood in a scattered manner, a concerted effort to include both asymmetries has so far not been attempted.

The structure of colloidal solutions and the surrounding small ions has been improved from time to time from the classical Poisson–Boltzmann (PB) description of point ions,^21^ to the modified Gouy–Chapman theory (MGC) for RPM and unequal radius MGC for PM, to include correlations arising due to different contact distances at the Stern layers.^31^ A number of studies over the years have been able to confirm that along with charges on the ions and the interface constituting the EDL, size correlations of ions as well as the solvent components also contribute quite substantially in deciding its static structure. These include liquid state analytical theories, *viz.* modified PB theory,^32^ integral equation theory,^33^ and density functional theory,^34^ and also simulation methods.^35–37^ In recent times, a number of delicate experiments also point to the detailed structural behavior of ionic clouds of double layers around colloidal macroions. These include atomic force microscopy (AFM),^38^ optical tweezer experiments,^39^ X-ray photoelectron spectroscopy,^40^ electrokinetic measurements,^41^ small angle X-ray scattering (SAXS),^35^*etc.* Attempts to include off-center charges of small ions rather than primitive centrally located ones have also been studied in great detail in recent times.^42^ In the current work, however, we restrict ourselves to the primitive models but include the solvent as an individual component in the mixed electrolyte system, to understand the effects of charge and size asymmetry within the same spherical double layer (SDL). Apart from an individual component description, the solvent is also included as a continuum with the dielectric constant of water. The theoretical formalism is presented in Section II, the results and discussion part is given in Section III, and finally we offer a few concluding remarks in Section IV.

## II. Theoretical formulation

### A. Solvent primitive model

**Fig. 1:**
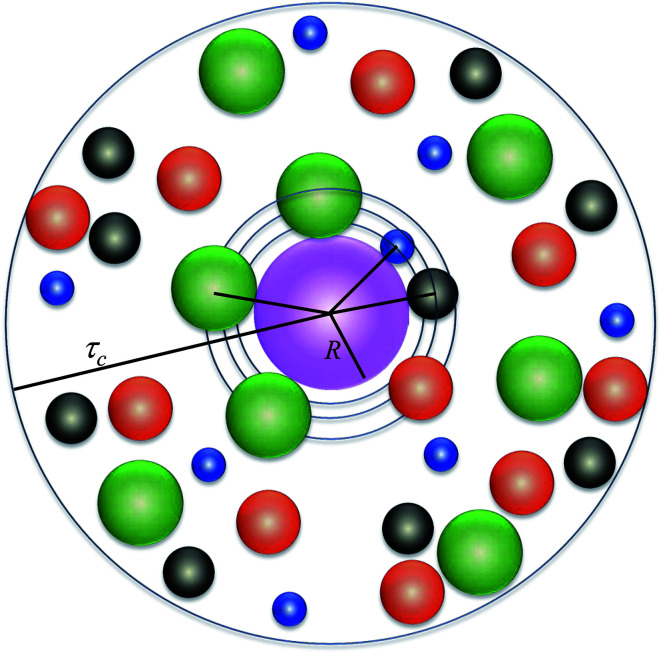
Schematic representation of the model system. The different colors correspond to blue: Mg^2+^, black: Na^+^, red: solvent molecules, and green: Cl^−^.

The system considered here consists of mixed electrolytes (NaCl/MgCl_2_) immersed in a solvent around an isolated uniformly charged spherical colloidal macroparticle of radius *R* with surface charge density (*Q*) written as1
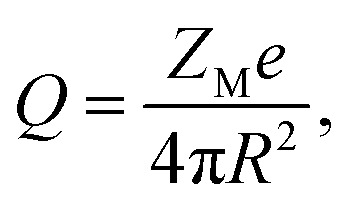
where *Z*_M_ is the valence of the macroion with *e* as the electronic charge. The solvent primitive model (SPM) is adopted here, where the electrolyte solution consists of the small ions and the solvent, that are taken as the charged and neutral hard spheres, respectively. The schematic representation of the model system is given in Fig. 1. The mixed electrolyte is taken as a mixture of 1 : 2 : 1 NaCl/MgCl_2_, with *z*_*α*_ as the valence of the ions. The electrolyte solution, therefore, consists of four components, *α* = 1–4, with diameters *σ*_*α*_, where the 4th component (*α* = 4) represents the solvent (*z*_*α*_ = 0). For simplicity, the diameter ratio is always taken as 1 : 1.25 : 2 : 1.5, which implies that, *σ*_1_ = *σ*, *σ*_2_ = 1.25*σ*, *σ*_3_ = 2*σ*, *σ*_4_ = 1.5*σ*, with the smallest ionic diameter (*σ*) equal to 0.2125 nm. The smallest ion is taken always as the multivalent one (Mg^2+^) justifying^44^ the use of bare ionic diameters rather than the hydrated one. The solvent diameter is taken as *σ*_4_ = 1.5*σ*, in between the counterion and coion, the reason being the difficulty of having a huge number of solvent molecules representing the bulk water. This is also the sole reason for taking the concentration of the solvent (*C*_S_) as 27.75 M instead of bulk water at 55.5 M. However, the steric interactions can still be envisaged even with this solvent concentration. At low electrolyte concentrations (0.1 M), it is possible to include bulk water (55.55 M) as the solvent. Alongside the solvent taken as a component, it still resembles water with dielectric constant *ε* as 78.5 at temperature, *T* = 298 K. It is to be noted here that with this single dielectric constant (78.5), the effects due to image charges are completely ignored, which may not be an exact situation considering different Stern layers formed due to the unequal size of the small ions.^43^ The interaction potential between any two components (*α* and *β*) of the electrolyte solution is given as2
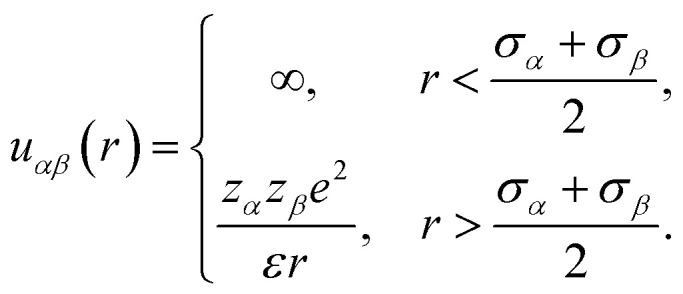
with the solvent–solvent and solvent–small ion interactions automatically tuned to hard-sphere potentials. Similarly, the interaction potential between any component of the electrolyte (*α*) and the macroion is written as3
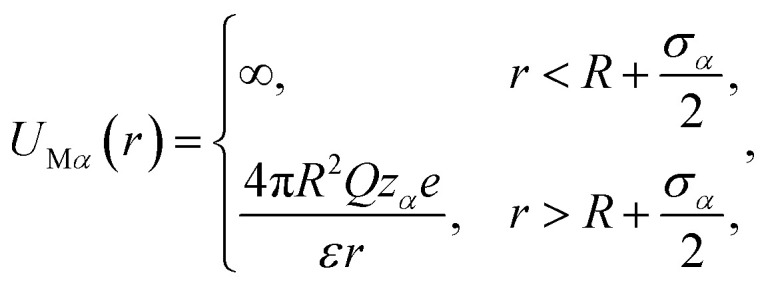
with the automatic representation of the solvent for *z*_*α*_ = 0.

### B. Density functional theory

In DFT,^34^ the grand potential, *Ω* or the free energy, *F* of the system is a functional of nonuniform density {*ρ*_*α*_(**r**)} and they are related to each other through a Legendre transform given as4
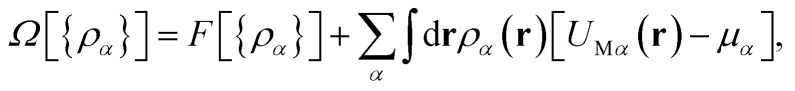
where {*ρ*_*α*_} is the singlet density distribution and *μ*_*α*_ is the chemical potential, for component *α*. This grand potential, *Ω* should be the minimum at equilibrium that gives rise to the final density profile of any component (*α*) in the present SDL written as

5 *ρ*_*α*_(*r*) = *ρ*^0^_*α*_ exp{−*β*_0_*z*_*α*_*ψ*(*r*) + *c*^(1) hs^_*α*_(*r*;[{*ρ*_*α*_}]) − *c*^(1) hs^_*α*_([{*ρ*^0^_*α*_}]) + *c*^(1) el^_*α*_(*r*;[{*ρ*_*α*_}]) − *c*^(1) el^_*α*_([{*ρ*^0^_*α*_}])},

where *ρ*^0^_*α*_ is the uniform counterpart, *β*_0_ = (*k*_B_*T*)^−1^, is the inverse temperature with *k*_B_ being the Boltzmann constant and *T* the temperature. In eqn (5), *c*^(1)^_*α*_(**r**;[{*ρ*_*α*_]) is the first-order correlation function, and *ψ*(**r**) represents the mean electrostatic potential (MEP) arising due to all the ionic charges, and can be written as^45^6
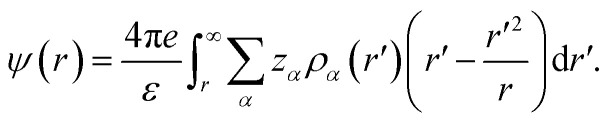


The expression for the density profile [eqn (5)], although exact, cannot be evaluated directly due to the unavailability of exact quantities of nonuniform *c*^(1)^_*α*_(*r*;[{*ρ*_*α*_]). Hence a number of approximations are attempted from time to time for the calculation of these quantities. Here, a partially perturbative procedure is adopted and the hard-sphere and electrical components are approximated through different routes. Here, the hard sphere part, *c*^(1) hs^_*α*_(**r**;[{*ρ*_*α*_}]) is evaluated through the Denton and Ashcroft (DA)^46^ weighted density approach (WDA) given by

7 *c*^(1)hs^_*α*_(**r**;[{*ρ*_*α*_}]) = *c̃*^(1)hs^_*α*_(*

<svg xmlns="http://www.w3.org/2000/svg" version="1.0" width="13.846154pt" height="16.000000pt" viewBox="0 0 13.846154 16.000000" preserveAspectRatio="xMidYMid meet"><metadata>
Created by potrace 1.16, written by Peter Selinger 2001-2019
</metadata><g transform="translate(1.000000,15.000000) scale(0.013462,-0.013462)" fill="currentColor" stroke="none"><path d="M320 1000 l0 -40 240 0 240 0 0 40 0 40 -240 0 -240 0 0 -40z M480 840 l0 -40 -80 0 -80 0 0 -120 0 -120 -40 0 -40 0 0 -80 0 -80 -40 0 -40 0 0 -200 0 -200 40 0 40 0 0 120 0 120 160 0 160 0 0 40 0 40 40 0 40 0 0 40 0 40 40 0 40 0 0 80 0 80 40 0 40 0 0 120 0 120 -40 0 -40 0 0 40 0 40 -120 0 -120 0 0 -40z m240 -120 l0 -80 -40 0 -40 0 0 -80 0 -80 -40 0 -40 0 0 -80 0 -80 -120 0 -120 0 0 80 0 80 40 0 40 0 0 120 0 120 40 0 40 0 0 40 0 40 120 0 120 0 0 -80z"/></g></svg>

*^(*α*)^(**r**)),

where, the DA prescription is used to calculate the weighted density, *ρ̄*_*α*_(**r**) representing the uniform fluid counterpart. The electrical contribution, *c*^(1)el^_*α*_(**r**;[{*ρ*_*α*_}]) is calculated through the perturbation route as,^47^8
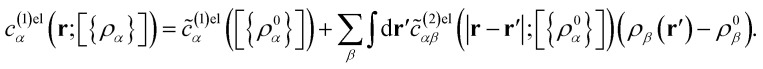


The direct correlation functions, *c̃*^(2)hs^_*αβ*_ and *c̃*^(2)el^_*αβ*_ required to calculate *c*^(1) hs^_*α*_ and *c*^(1) hs^_*α*_, [eqn (7) and (8)] are taken from the analytical expressions as given by Blum^48^ and Hiroike^49^ within the mean spherical approximation (MSA) of the electrolyte solution for size and charge asymmetric mixtures of neutral and charged hard spheres. The calculation of density and the MEP profiles are now quite obvious from eqn (5) and (6) using an iterative solution method.

### C. Monte Carlo simulations

In the current work, the model system is simulated through canonical Monte Carlo (CMC) simulations (*N*, *V*, *T*). The initial configuration is generated by fixing the macroion at the center of a cubic simulation cell and inserting the small ions and the solvent to attain the desired concentration. The cell is sufficiently large so that the interactions with any other cell can be neglected.^50^ Considering the spherical symmetry of the problem, in all three perpendicular directions (*X*, *Y*, and *Z*), the periodic boundary conditions are employed. Once the initial configuration is generated, the system is equilibrated through random diffusion of the components in random translational moves. The long range potential between the charges is taken care of through the Ewald summation.^51^ The standard Metropolis sampling procedure^52^ is adopted for acceptance of the moves. A block averaging procedure is used and the density profile is calculated by dividing the box into spherical bins, and the MEP profiles from the respective component densities. The relevance of the simulation is justified with the assumptions based on the model as it will be exact for the given model. For example, the size correlations for individual components were thought to be better manifested in the current method as it resembles hard-sphere mixtures in a spherical geometry. Similarly, the charge correlations will be clearly visualized, once the size correlations are taken out. The beauty of the present simulation also lies in partitioning the contribution of hard-sphere solvent in the overall size and charge correlations.

## III. Results and discussion

The main strategy behind the current work is to look at the size and charge correlations that are effected in the components that constitute the electrolyte solution on the structure of the SDL, around a colloidal macroion within the SPM representation. To make things more general, an individual component representation of the solvent is considered along with the mixed electrolyte system. The singlet density profiles of the small ions and the solvent in the SDL for the mixed salt, *i.e.*, 1 : 2 : 1 (NaCl/MgCl_2_) with 1 M 1 : 1 (NaCl) electrolyte at varying concentrations of 2 : 1 (MgCl_2_) salt, are calculated. Unless otherwise stated, the macroion surface charge density is fixed at *Q* = 0.102 C m^−2^, with the radius kept constant at *R* = 1.5 nm. Alongside a wide variation of macroion radii, the radius of coions, the surface charge density on the macroion, and the concentration of the electrolyte and that of the solvent, are also varied to test the effect of all the parameters that influence the properties of double layers in direct or indirect ways. This also allows direct comparison on DFT and MC predictions to widen their horizons within the SDL.

**Fig. 2:**
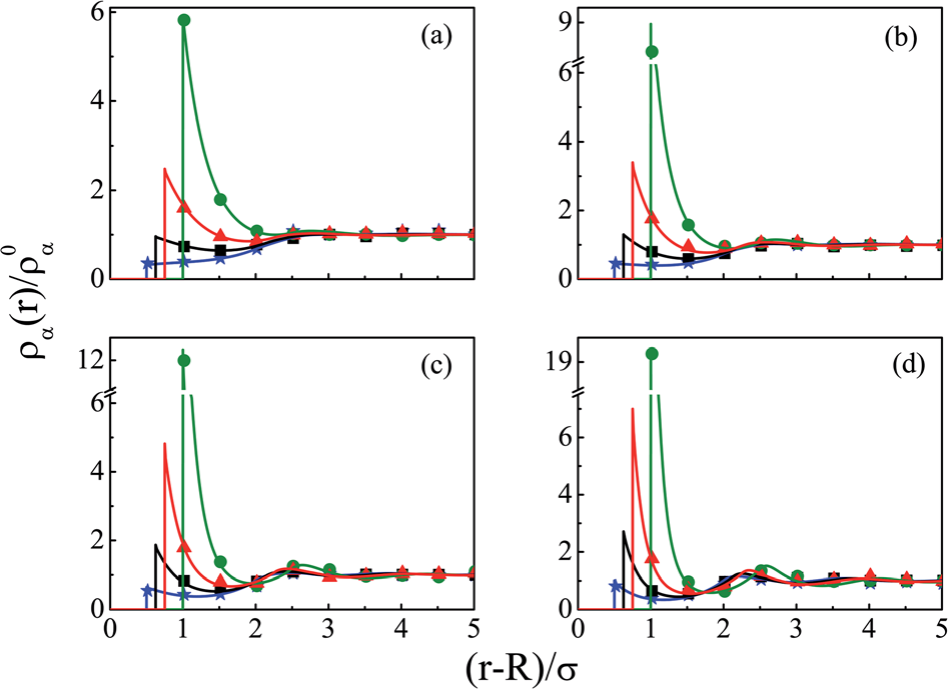
Component density profiles (small ions and solvent) around a colloidal macroion of *R* = 1.5 nm with *Q* = 0.102 C m^−2^ in a mixed electrolyte solution of 1 M NaCl and 0.5 M MgCl_2_ with different solvent concentrations: (a) 15 M, (b) 20 M, (c) 25 M, and (d) 30 M. The symbols and solid lines represent simulation and DFT results, respectively. The different lines and symbols correspond to blue, ⋆: Mg^2+^, black, □: Na^+^, red, △: solvent molecules, and green, ○: Cl^−^.

**Fig. 3:**
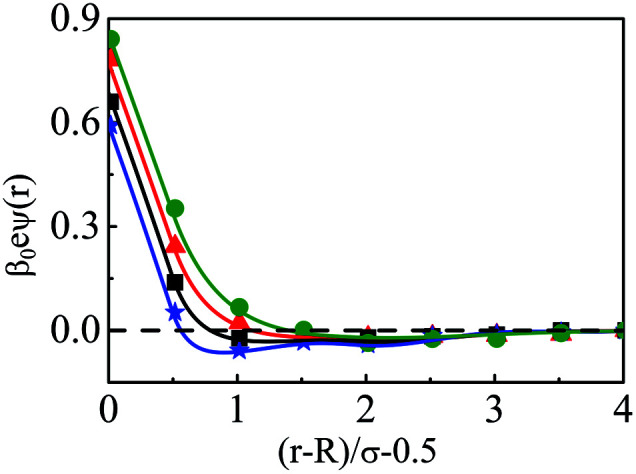
Mean electrostatic potential profiles around a colloidal macroion of *R* = 1.5 nm with *Q* = 0.102 C m^−2^ in a mixed electrolyte solution of 1 M NaCl and 0.5 M MgCl_2_ with different solvent concentrations: (a) 15 M (green, ○), (b) 20 M (red, △), (c) 25 M (black, □), and (d) 30 M (blue, ⋆). The symbols and solid curves represent simulation and DFT results, respectively.

The density profiles of individual components of an SDL having supporting electrolytes, 1 M NaCl with 0.5 M MgCl_2_ around a macroion at *Q* = 0.102 C m^−2^ of *R* = 1.5 nm, are depicted in Fig. 2, with the variation of concentration of solvent (*C*_S_) from (a) 15 M to (d) 30 M. As expected, the density profiles of all the components show increasing oscillations in moving from lower to higher solvent concentrations. This is the packing entropic effect that dominates with an increase in the total number of molecules. Size correlation is also reflected in the multiple layer formation. The separation of charges among the ionic components is manifested with the increasing thickness. Also the double layer shows sufficient increase in its width from the macroion that attends the bulk values of the component densities. At low solvent concentration (*C*_S_), the coion density at contact is lower, however, this also started increasing with increasing solvent concentration, indicating the OC effect in moving from the inner Helmholtz planes (IHPs) that start at (*R* + *σ*_*α*_/2) for *α* = 1, 2, to the outer Helmholtz plane (OHP) starting at (*R* + *σ*_3_/2). As is evident, the CR effect starts appearing at the lowest solvent concentration (15 M), and becomes more pronounced with increasing solvent concentration. A clearer picture for this emerges as we plot the MEP profiles with variation in solvent concentrations. Thus, Fig. 3, depicts a faster rate of drop of MEP towards the CR phenomena at higher solvent concentrations. A critical examination on the MEP curve for solvent concentration, *C*_S_, at 30 M shows substantial oscillations indicating multiple CR effects. This also confirms the larger width of the double layer for higher solvent concentrations.

**Fig. 4:**
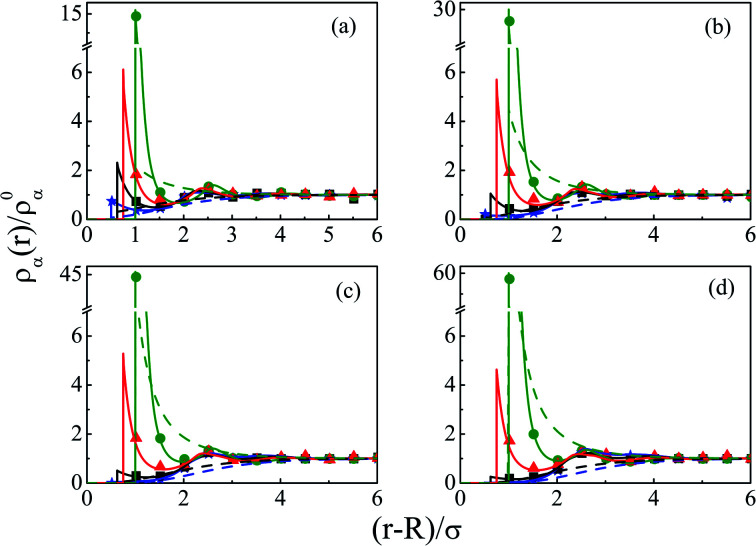
Component density profiles (small ions and solvent) around a colloidal macroion of *R* = 1.5 nm in a mixed electrolyte solution of 1 M NaCl and 0.5 M MgCl_2_ with fixed solvent concentration (*C*_S_) of 27.75 M, and at various surface charge densities: (a) *Q* = 0.102 C m^−2^, (b) *Q* = 0.204 C m^−2^, (c) *Q* = 0.306 C m^−2^, and (d) *Q* = 0.408 C m^−2^. The symbols, solid and dashed curves represent simulation, DFT, and URMGC results, respectively.

**Fig. 5:**
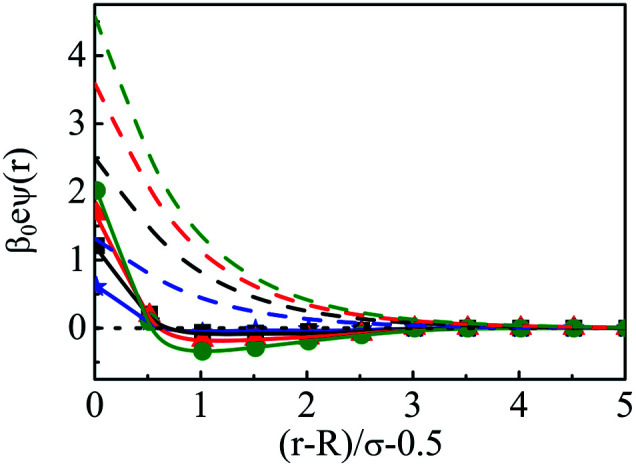
Mean electrostatic potential profiles around a colloidal macroion of *R* = 1.5 nm in a mixed electrolyte solution of 1 M NaCl and 0.5 M MgCl_2_ with fixed solvent concentration (*C*_S_) of 27.75 M, and at various surface charge densities: (a) *Q* = 0.102 C m^−2^ (blue, ⋆), (b) *Q* = 0.204 C m^−2^ (black, □), (c) *Q* = 0.306 C m^−2^ (red, △), and (d) *Q* = 0.408 C m^−2^ (green, ○). The symbols, solid and dashed curves represent simulation, DFT, and URMGC results, respectively.

As we move on to the variation of other important parameters at constant solvent concentration of *C*_S_ = 27.75 M, for the current SDL, a number of interesting features started appearing in terms of OC and CR effects. Thus, Fig. 4 depicts the density distributions of all the components around the macroion for a mixture of 1 M NaCl and 0.5 M MgCl_2_ with varying *Q* from (a) 0.102 to (d) 0.408 C m^−2^. Also plotted are the URMGC ionic profiles, that show that the profiles are completely monotonic with lower double layer thickness as compared to DFT or MC predictions. As is clear, the counterions are strongly attracted and the coions get repelled at the interface. With increasing *Q*, this is seen clearly, as even the solvent density at the interface starts decreasing along with the coions. This is because of the larger size of the counterion, that gets enhanced with the opposite charge. In fact, the OC phenomena starts disappearing when moving towards higher *Q*. Although the spread of the double layer is reflected by an increase in the thickness during passage to higher *Q*, the width remains more or less constant. The density profiles also point to the onset of charge inversion from low *Q*, which starts on increasing with higher *Q*. The same has become quite clear in the MEP profiles depicted in Fig. 5, which showed an increased diffuse layer potential at contact. The rate of drop of MEP into the CR zone is quite faster at higher *Q*, although the variation becomes slower in the end, thereby pointing to the same width even at higher *Q*. The growth of thickness is also reflected in the MEP which is due to the continuous decrease of coion density together with increased counterion density at contact.

**Fig. 6:**
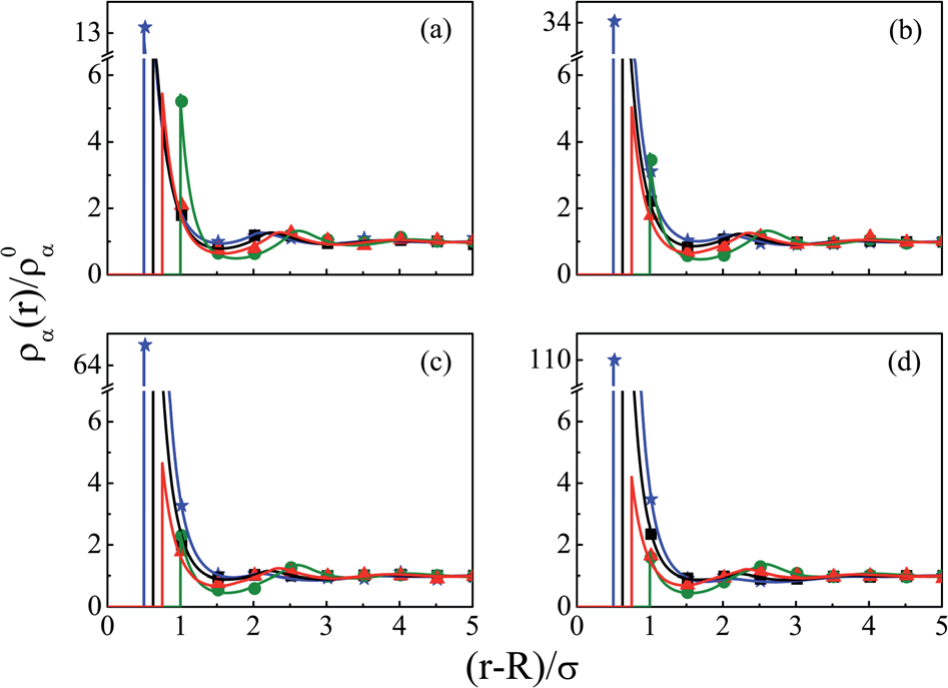
Component density profiles (small ions and solvent) around a colloidal macroion of *R* = 1.5 nm in a mixed electrolyte solution of 1 M NaCl and 0.5 M MgCl_2_ with fixed solvent concentration (*C*_S_) of 27.75 M, and at various surface charge densities: (a) *Q* = −0.102 C m^−2^, (b) *Q* = −0.204 C m^−2^, (c) *Q* = −0.306 C m^−2^, and (d) *Q* = −0.408 C m^−2^. The symbols and solid curves represent simulation and DFT results, respectively.

**Fig. 7:**
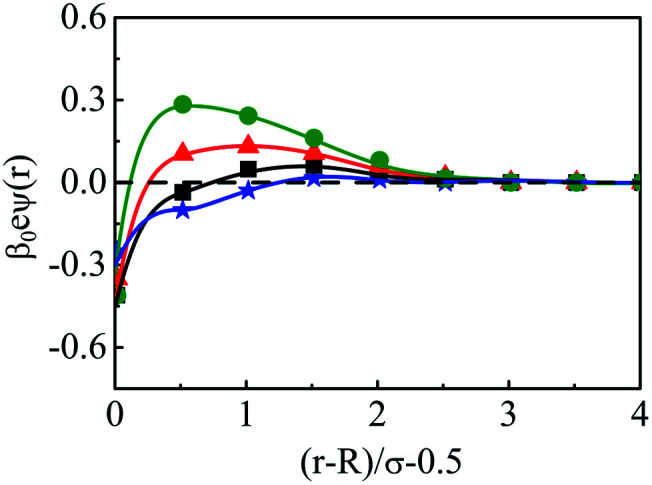
Mean electrostatic potential profiles around a colloidal macroion of *R* = 1.5 nm in a mixed electrolyte solution of 1 M NaCl and 0.5 M MgCl_2_ with fixed solvent concentration (*C*_S_) of 27.75 M, and at various surface charge densities: (a) *Q* = −0.102 C m^−2^ (blue, ⋆), (b) *Q* = −0.204 C m^−2^ (black, □), (c) *Q* = −0.306 C m^−2^ (red, △), and (d) *Q* = −0.408 C m^−2^ (green, ○). The symbols and solid curves represent simulation and DFT results, respectively.

Since the larger sized counterions always lead to a substantial increase in density at the interface on the macroion, it would be worthwhile seeing what the effect of the larger coion would be on the same system. Thus, Fig. 6 depicts a multiple increase in the contact density of Mg^2+^, when the individual density profiles are plotted for a mixture of 1 M NaCl and 0.5 M MgCl_2_ with varying *Q* from (a) −0.102 to (d) −0.408 C m^−2^. This is because of its higher valence coupling with the smaller size of the coion. Although the coion density profiles show gradual decrease in moving to higher *Q*, the contact density is still quite large because of the higher concentration of the coion (Cl^−^). However, the solvent density shows a gradual decrease because its size is between that of the coion and the counterions. The separation of charges becomes quite large and manifests through the increased thickness, although the dampening of the system within a short distance leads to a contraction of the double layer in its width. The same is also reflected in the MEP profiles as shown in Fig. 7, where the rate of drop of potential is quite steep in passing to the larger *Q*, indicating passage to the CR area quite fast. It is also quite clear from the MEP profiles that the CR effect started appearing from a lower *Q* itself.

**Fig. 8:**
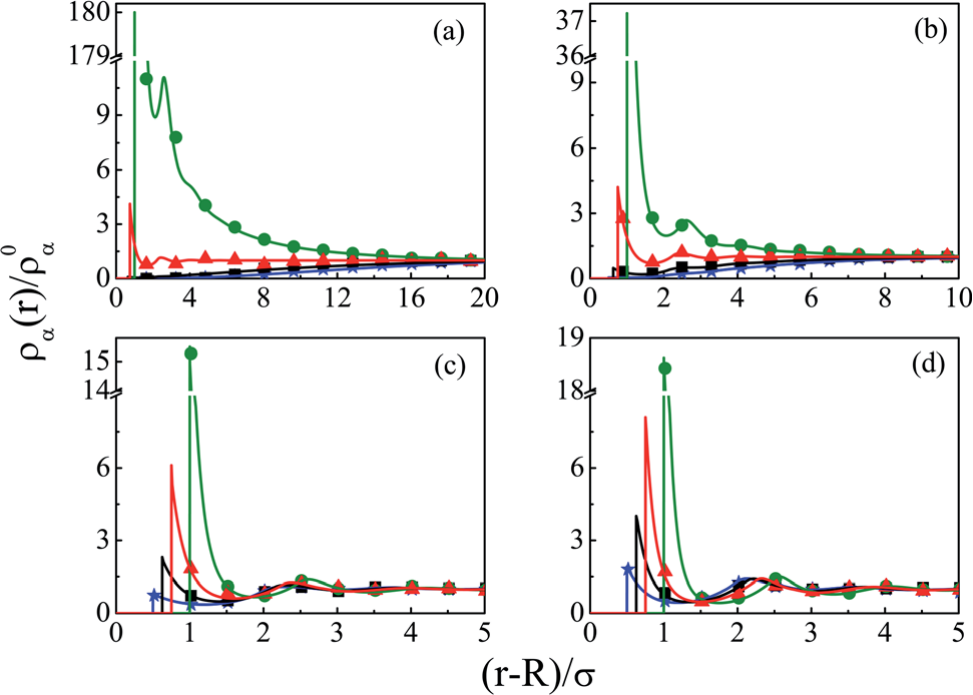
Component density profiles (small ions and solvent) around a colloidal macroion of *R* = 1.5 nm with *Q* = 0.102 C m^−2^ in a mixed electrolyte solution at fixed solvent concentration (*C*_S_) of 27.75 M and with various electrolyte concentrations: (a) 0.01 M NaCl and 0.005 M MgCl_2_, (b) 0.1 M NaCl and 0.05 M MgCl_2_, (c) 1 M NaCl and 0.5 M MgCl_2_, and (d) 2 M NaCl and 1 M MgCl_2_. The symbols and solid curves represent simulation and DFT results, respectively.

**Fig. 9:**
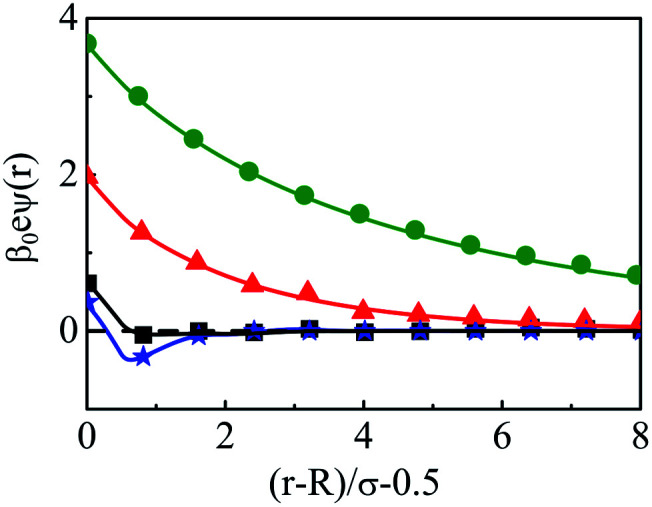
Mean electrostatic potential profiles around a colloidal macroion of *R* = 1.5 nm with *Q* = 0.102 C m^−2^ in a mixed electrolyte solution at fixed solvent concentration (*C*_S_) of 27.75 M and with various electrolyte concentrations: (a) 0.01 M NaCl and 0.005 M MgCl_2_ (green, ○) (black, □), (b) 0.1 M NaCl and 0.05 M MgCl_2_ (red, △), (c) 1 M NaCl and 0.5 M MgCl_2_ (black, □), and (d) 2 M NaCl and 1 M MgCl_2_ (blue, ⋆). The symbols and solid curves represent simulation and DFT results, respectively.

In order to have a clearer picture of the contribution of charge correlations in the current SDL, we move on to increase the concentration of the electrolyte, whereas the concentration of the solvent still remains constant at *C*_S_ = 27.75 M. Thus, Fig. 8 depicts the component density profiles for a mixture of NaCl and MgCl_2_ where the concentration is varied from 0.01 M to 2 M by keeping the ratio of NaCl : MgCl_2_ = 2 : 1, around a macroion of *R* = 1.5 nm, with *Q* = 0.102 C m^−2^. As expected, the counterion density profile shows a lot of enhancement due to the electrostatic attraction and the opposite effect becomes true for the coions. The volume exclusion becomes quite prominent with an increase in the number of layers as we pass on to higher electrolyte concentrations. Charge correlations started contributing leading to an increase in OC phenomena. The interplay between charge and size correlations also leads to an increase in solvent density at the interface. The dampening of densities and the CR effect starts from 1 M electrolyte concentration. The same is also corroborated through the MEP profiles, as depicted in Fig. 9, where it is quite clear that there is a double inversion for 2 M electrolyte concentrations. As far as the spread of SDL goes, it is quite clear that the width and thickness of the double layer continuously decreases in moving towards higher electrolyte concentrations because of effective screening of the charge on the macroion by the individual components of the electrolyte.

**Fig. 10:**
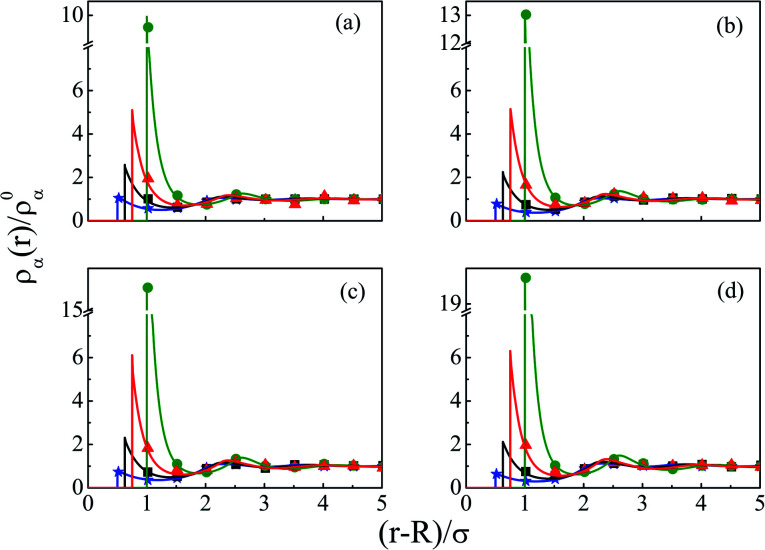
Component density profiles (small ions and solvent) of a mixed electrolyte solution of 1 M NaCl and 0.5 M MgCl_2_ at *C*_S_ of 27.75 M, around a spherical macroion with *Q* = 0.102 C m^−2^ and different radii: (a) *R* = 0.5 nm, (b) *R* = 1 nm, (c) *R* = 1.5 nm, and (d) *R* = 6 nm. The symbols and solid curves represent simulation and DFT results, respectively.

**Fig. 11:**
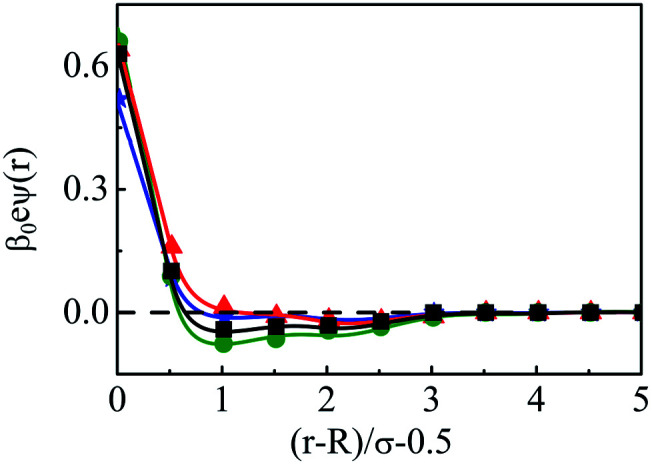
Mean electrostatic potential profiles of a mixed electrolyte solution of 1 M NaCl and 0.5 M MgCl_2_ at *C*_S_ of 27.75 M, around a spherical macroion with *Q* = 0.102 C m^−2^ and having different radii: (a) *R* = 0.5 nm (blue, ⋆), (b) *R* = 1 nm (black, □), (c) *R* = 1.5 nm (red, △), and (d) *R* = 6 nm (green, ○). The symbols and solid curves represent simulation and DFT results, respectively.

The other important parameter through which both the charge and size correlations can be effected is the size of the macroion, hence, it is varied from (a) *R* = 0.5 nm to (d) *R* = 6 nm, by keeping the other parameters the same. Thus, Fig. 10 depicts the component density profiles for the SDL. In fact, this is also an attempt to study the effect of increasing the overall macroion charge on the SDL. As is clear, there is a slow decrease of coion densities and the same rate of increase of counterion densities at the interface. This is on the expected line as the large sized counterions will be accommodated more compared to small sized coions. In fact, that is the case for solvent density also, which shows a slow increase in its contact density in moving to the case of a large sized macroion. This also leads to substantial increases in layering with multiple layers. The OC effect tends to have a slow decrease and there should be a slow increase in the CR effect. The same effects are also reflected in the MEP profiles plotted in Fig. 11. There is a sharp drop of the MEP for the larger sized macroion due to the increased CR effect. As far as the spread of the SDL is concerned, the thickness goes on continuously increasing, although the width remains more or less constant.

**Fig. 12:**
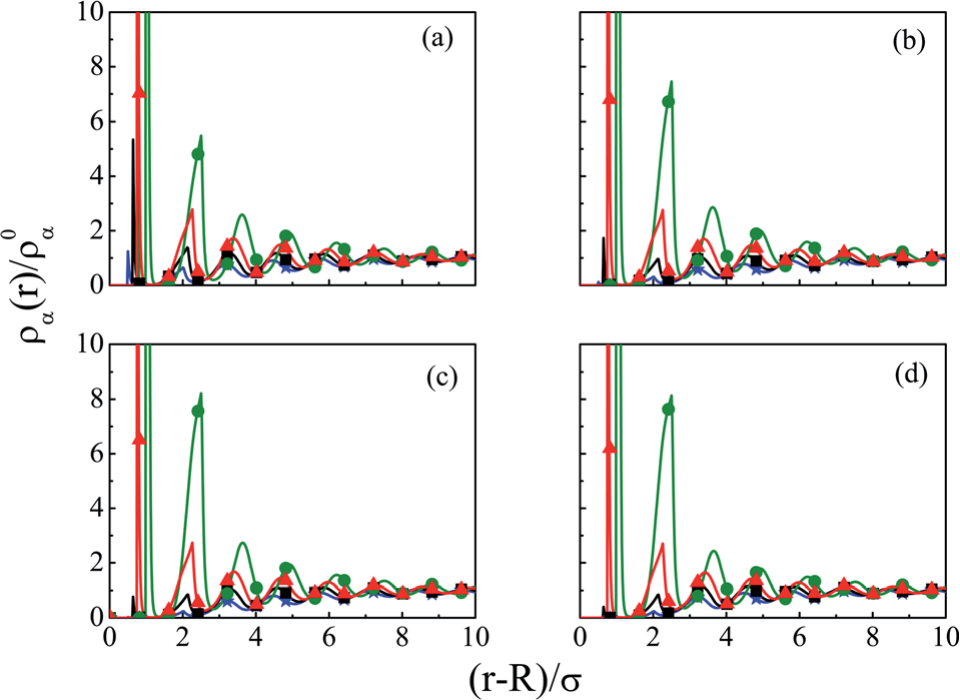
Component density profiles (small ions and solvent) around a colloidal macroion of *R* = 1.5 nm in a mixed electrolyte solution of 0.1 M NaCl and 0.05 M MgCl_2_ with fixed solvent concentration (*C*_S_) of 55.55 M, and at various surface charge densities: (a) *Q* = 0.102 C m^−2^, (b) *Q* = 0.204 C m^−2^, (c) *Q* = 0.306 C m^−2^, and (d) *Q* = 0.408 C m^−2^. The symbols, solid and dashed curves represent simulation, DFT, and URMGC results, respectively.

**Fig. 13:**
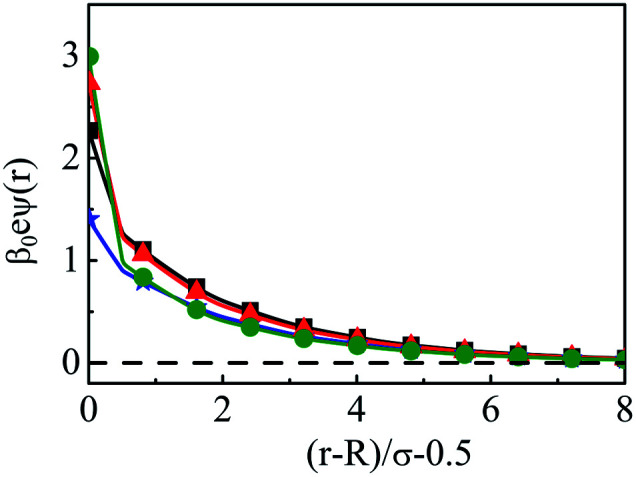
Mean electrostatic potential profiles around a colloidal macroion of *R* = 1.5 nm in a mixed electrolyte solution of 0.1 M NaCl and 0.05 M MgCl_2_ with fixed solvent concentration (*C*_S_) of 55.55 M, and at various surface charge densities: (a) *Q* = 0.102 C m^−2^ (blue, ⋆), (b) *Q* = 0.204 C m^−2^ (black, □), (c) *Q* = 0.306 C m^−2^ (red, △), and (d) *Q* = 0.408 C m^−2^ (green, ○). The symbols, solid and dashed curves represent simulation, DFT, and URMGC results, respectively.

As steric effects due to the solvent play a crucial role in determining the ionic distributions as well as OC and CR effects, it will be highly desirable to include the bulk water concentration (55.55 M), instead of a lower value. However, this is possible only at a lower concentrations of the electrolyte. Thus, Fig. 12 depicts the density profiles of the components at *C* = 0.1 M and *C*_S_ = 55.5 M at different *Q*, as they vary away from the surface. Since the solvent concentration is quite high, the oscillations seems to be quite large and take a longer distance to diffuse. Both the width as well as the thickness keeps on increasing with increasing *Q*. The diffusive behavior of the double layers is also envisaged in the MEP profiles as can be seen from Fig. 13, where the MEP drops to zero at a large distance from the macroion.

**Fig. 14:**
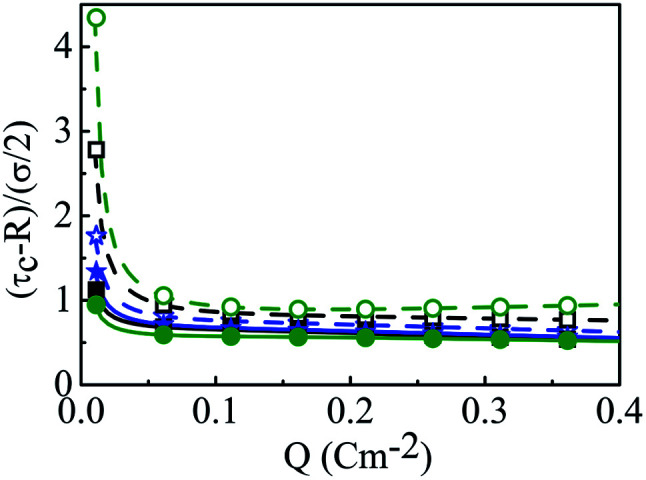
Capacitive compactness values (*τ*_c_) as a function of surface charge density *Q* (C m^−2^) around a colloidal macroion of *R* = 1.5 nm in a mixed electrolyte solution of 1 M NaCl and 0.5 M MgCl_2_, with solvent concentration (*C*_S_) of 27.75 M (solid lines), and without solvent (dashed lines), and with different small ion (Mg^2+^) diameters: (a) *σ* = 0.1 nm (blue), (b) *σ* = 0.15 nm (black), and (c) *σ* = 0.2125 nm (green). The symbols and solid curves represent simulation and DFT results, respectively.

A critical measurement of the width of the double layer is quite important to calculate the spread and the associated influence of the macroion charge and is defined through the capacitive compactness, given as^53,54^9
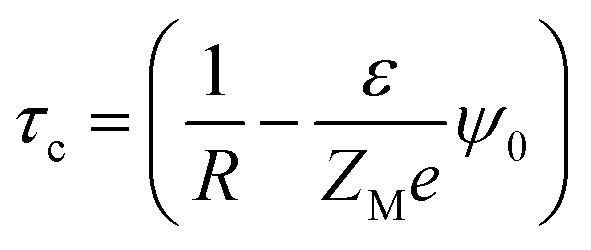
where *ψ*_0_ is the MEP at the macroion surface. The capacitive compactness (*τ*_c_) data is plotted in Fig. 14 against the colloidal charge density (*Q*) for an SDL having supporting electrolytes of 1 M NaCl with 0.5 M MgCl_2_ around a macroion of *R* = 1.5 nm, with and without the solvent, with different small ion sizes. In the absence of solvent, on decreasing the small ion size, the double layer becomes more compact. The trend is the exact opposite, in the presence of solvent. However, the compactness of the double layer is reduced in the presence of the solvent.

The other important quantity that can be accessed through experiment^40^ is the integrated charge distribution function *P*(*r*), that represents the net charge of the SDL centred around the macroion within the sphere of radius *r*, and is given as10
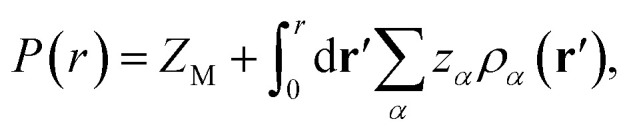


**Fig. 15:**
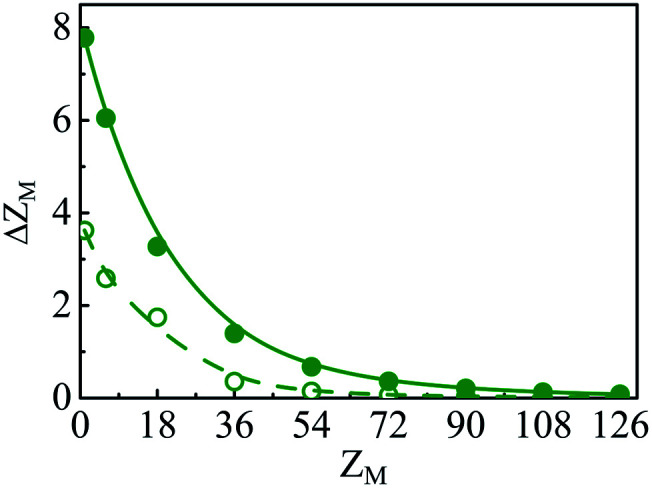
Adsorbed charge amplification (Δ*Z*_M_) as a function of *Z*_M_ for a colloidal macroion of *R* = 1.5 nm in a mixed electrolyte solution of 1 M NaCl and 0.5 M MgCl_2_, with solvent concentration (*C*_S_) of 27.75 M (solid line), and without solvent (dashed line). The symbols and solid curves represent simulation and DFT results, respectively.

In essence, *P*(*r*)*Z*_M_ < 0 indicates the CR effect, whereas, *P*(*r*)*Z*_M_ > 0 or |*P*(*r*)| > |*Z*_M_| corresponds to surface charge amplification. The charge amplification as defined through the difference of maximum *P*(*r*) and that of the bare macroion charge (*Z*_M_), given as Δ*Z*_M_ = *P*(*r*)_max_ − *Z*_M_ is plotted in Fig. 15 against *Z*_M_ for the SDL with 1 M NaCl and 0.5 M MgCl_2_ around a macroion of *R* = 1.5 nm, with and without the solvent. It is quite clear that the adsorbed charge amplification increased quite drastically in the presence of solvent. The results obtained from DFT can be quantitatively reproduced through simulations. It should be noted here that experiments based on surface potential measurements and the Helmholtz layer composition can be planned on nanoporous silica or polystyrene particles in the presence of supporting electrolyte solutions that includes solvent. However, specific interpretations for the adsorbed surface charges can be ascertained by coupling molecular modeling with the experimental characterization.^55^

## IV. Concluding remarks

The major emphasis of the present work is to investigate in detail the charge and size correlations that arise due to the combined effect or charge and size asymmetry on the SDL formed from a mixed electrolyte solution that includes the solvent as an individual component. As a matter of fact, the electrolyte solution consists of four components of different diameters, with a multivalent coion and a neutral solvent. The solvent also makes its presence known as a dielectric medium through the interaction reduced for the bare ions. For simplicity, the solvent is taken as that of water through its dielectric constant, which, however, was kept at the concentration (*C*_S_) at half of that of bulk water at 27.75 M due to constraints in the simulations. However, for the low electrolyte concentration (0.1 M), bulk water concentration can also be included. The DFT adopted here is a partially perturbative scheme, where the DA version^46^ of the WDA is used to calculate the hard-sphere part of the free energy contribution and the ionic part through the perturbation with respect to the bulk density. The required DCF to evaluate the hard sphere and the ionic part is taken from the work of Blum^48^ and Hiroike^49^ on multicomponent electrolytes. For comparison, a canonical ensemble Monte Carlo simulation is also performed on the same system. A wide variation of different parameters are attempted to throw light on the overcharging and charge reversal phenomena.

The size correlations in the SDL is directly reflected in the increase of solvent concentration (*C*_S_) that indicates the increase in volume exclusion, formation of multiple numbers of layers, increase of charge separation, and the widening of the charge reversal effects. The increase of thickness of the double layer is also visible in increasing macroion surface charge density (*Q*), although there is hardly any change in its width. Increasing the coion size larger than the counterion leads to a quick drop of MEP to the CR area within a short distance from the interface indicating a clear increase in the charge separation. This also leads to a decrease in the width of the SDL. The importance of charge correlation is reflected through the increase in concentration of the electrolyte, where the OC effect as well as the CR effect shows a gradual increase. This leads to a decrease of the width of the double layer leading to the dampening of the density profiles. However, increasing the overall charge on the macroion (*Z*_M_) by increasing the size of the macroion, leads to a slow decrease of the OC effect and a slow increase in the CR effect. In all the parametric variations, it is quite clear that the density and the MEP profiles show a quantitative comparison for both the DFT and MC results. The capacitive compactness data clearly indicates that the presence of solvent reduces the compactness of the double layer studied here.

The current study concentrates on the effect of charge and size asymmetry by including solvent in the mixed electrolyte system, that is, having a multivalent cation. Although, a simplistic solvent primitive model with a reduced solvent density than that of the bulk molarity of water is considered here, a rigorous representation^56^ of the solvent along with the electrolyte ions is also within the purview of the present study. This description can also be applied extensively to any type of symmetry of the charged interface. In all the results presented here, only one dielectric constant as that of water (*ε* = 78.5) is considered here. However, due to the presence of three Helmholtz planes, the dielectric constant should not be the same everywhere.^43^ Extension of the same model to include image charges will be considered in the next study. The off-centric charge on the small ions will be a simple extension of the current study.^42^ The interaction between large colloidal particles can be normalized in terms of solvent, once the SDLs formed from two macroparticles can be considered. The importance of charge and size correlation in such solvent-induced interactions will be quite critical to numerically simulate biological macromolecules^57^ and even fuel cells^58^ and batteries.^59^ The current simulation method can also be applied to predict the Stern layer structure and energetics as accessed through synchrotron X-ray reflectivity (XRR) experiments^60^ for alkali chloride solutions on mica surfaces. Progress of all this work related to double layers will be reported in future publications.

## Conflicts of interest

There are no conflicts to declare.

## Supplementary Material
